# Genome Sequence of Type Strains of Genus *Stenotrophomonas*

**DOI:** 10.3389/fmicb.2016.00309

**Published:** 2016-03-10

**Authors:** Prashant P. Patil, Samriti Midha, Sanjeet Kumar, Prabhu B. Patil

**Affiliations:** Bacterial Genomics and Evolution Laboratory, Council of Scientific and Industrial Research – Institute of Microbial TechnologyChandigarh, India

**Keywords:** *Stenotrophomonas*, Type Strains, phylogenomics, Average Nucleotide Identity (ANI), evolution

## Abstract

Genomic resource of type strains and historically important strains of genus *Stenotrophomonas* allowed us to reveal the existence of 18 distinct species by applying modern phylogenomic criterions. Apart from *Stenotrophomonas maltophilia, S. africana* represents another species of clinical importance. Interestingly, *Pseudomonas hibsicola, P. beteli*, and *S. pavani* that are of plant origin are closer to *S. maltophilia* than the majority of the environmental isolates. The genus has an open pan-genome. By providing the case study on genes encoding metallo-β-lactamase and Clustered Regularly Interspaced Short Palindrome Repeats (CRISPR) regions, we have tried to show the importance of this genomic dataset in understanding its ecology.

## Background

The members of the genus *Stenotrophomonas* are widespread in the diverse habitats with biotechnological applications and clinical relevance (Ryan et al., 2009). According to a recent report by WHO, *S. maltophilia* is a leading drug-resistant pathogen in hospitals worldwide (Brooke, 2014). Currently, the genus *Stenotrophomonas* compromises 12 validated species in the List of Prokaryotic Names Standing in Nomenclature^1^ from diverse habitats. The type species of the genus *S. maltophilia* was initially isolated from the pleural fluid and named as *Bacterium bookeri* and was then reclassified as *P. maltophilia* (Hugh and Ryschenkow, 1961). Further, this species was transferred to genus *Xanthomonas* as *X. maltophilia* (Swings et al., 1983) and later it was designated as a distinct and new genus *Stenotrophomonas* (Palleroni and Bradbury, 1993). Remaining 11 species of this genus were isolated from distinct environmental sources, *S. rhizophila* (Wolf et al., 2002), *S. pavanii* (Ramos et al., 2011), *S. humi, S. terrae* (Heylen et al., 2007), *S. ginsengisoli* (Kim et al., 2010), and *S. panacihumi* (Yi et al., 2010), *S. koreensis* (Yang et al., 2006), *S. nitritireducens* (Finkmann et al., 2000), *S. acidaminiphila* (Assih et al., 2002), *S. chelatiphaga* (Kaparullina et al., 2009), and *S. daejeonensis* (Lee et al., 2011). Additionally, there are many taxonomical revisions in the genus *Stenotrophomonas*. *S. africana* was initially described as a novel species of this genus, isolated from human cerebrospinal fluid (Drancourt et al., 1997). But later based on the whole cell protein and DNA–DNA hybridization analysis *S. africana* was proposed as a synonym of *S. maltophilia* (Coenye et al., 2004). *S. dokdonesis* (Yoon et al., 2006) a former species of the genus *Stenotrophomonas*, was assigned to the new genus *Pseudoxanthomonas* (Lee et al., 2008). Apart from this, there are three species of genus *Pseudomonas*, i.e., *P. beteli, P. hibiscicola*, and *P. geniculata* which are transferred to the genus *Stenotrophomonas* considered as synonyms of the *S. maltophilia* (Van den Mooter and Swings, 1990; Anzai et al., 2000). *P. pictorium*, is also considered to be closer to *Stenotrophomonas* (Svensson-Stadler et al., 2012).

Herein we generated draft genomes of 16 type strains which include 11 currently validated species of the genus *Stenotrophomonas* and five genomes from the different genera which are historically associated or grouped with the *Stenotrophomonas*. Whole genome sequence of type strains of the *S. rhizophila* and *P. hibisicola* are available in the public database (**Table 1**). The complete genome of *S. maltophilia* type strain is available publically (Davenport et al., 2014), but we had also sequenced independently and hence included in this study. The genome sequence of the type strains will be valuable in taxonomic and evolutionary studies of genus *Stenotrophomonas* and its relatives.

**Table 1:** Genome features of the *Stenotrophomonas* genomes under study.

S. No	Species	Genome size (Mbp)	No. of contigs	Coverage fold	N50 (bp)	GC Content (%)	No. of CDS	Isolation source	Accession no.	Reference
1	*S. maltophilia* ATCC 13637^T^	4.98921	1	417	4989212	66.1	4645	Blood	CP008838	Davenport et al., 2014
2	*S. maltophilia* MTCC 434^T^	4.88156	306	52.5	41743	66.20	4327	Blood	JALV00000000	This study
3	*S. africana* LMG22072^T^	4.51217	173	116	47895	66.30	3991	Cerebrospinal fluid	LLXW00000000	This study
4	*P. hibsicola* ATCC 19867^T^	4.42403	20	NA	411451	66.40	3928	Plant	ARNB01000000	DOE-Joint Genome Institute^∗^
5	*P. beteli* LMG00978^T^	4.48462	109	184	83392	66.80	3907	*Piper betle*	LLXV00000000	This study
6	*S. pavanii DSM 25135*^T^	4.3138	129	115	79654	67.40	3783	Stem of sugarcane	LDJN00000000	This study
7	*P. geneculata* JCM*13324*^T^	4.80979	170	154	55144	66.20	4339	Tap water	LLXT00000000	This study
8	*S. chelatiphaga* DSM 21508^T^	3.96773	148	115	51682	66.90	3366	Municipal sewage	LDJK00000000	This study
9	*S. rhizophilia* DSM 14405^T^	4.64898	8	38	736911	67.30	3928	Rhizosphere soil	CP007597	Alavi et al., 2014
10	*S. panacihumi* JCM 16536^T^	3.92315	141	203	56105	68.80	3403	Soil	LLXU00000000	This study
11	*S. koreensis* DSM 17805^T^	3.0299	58	185	188329	66.10	2662	Compost	LDJH00000000	This study
12	*S. ginsengisoli* DSM24757^T^	3.37411	99	157	85121	65.90	2928	Field Soil	LDJM00000000	This study
13	*S. acidaminiphila JCM13310*^T^	3.94252	126	116	71728	68.80	3405	Sewage bioreactor	LDJO00000000	This study
14	*S. daeonesis* JCM *16244*^T^	3.28486	124	154	46611	68.60	2816	Sewage	LDJP00000000	This study
15	*P. pictorium* JCM 9942^T^	3.508292	84	193	89339	66.00	3099	Soil	LLXS00000000	This study
16	*S. humi* DSM 18929^T^	4.12205	92	143	171493	64.00	3549	Soil	LDJI00000000	This study
17	*S. nitritireducens* DSM 12575^T^	3.98349	95	140	167790	68.30	3387	Bio filters	LDJG00000000	This study
18	*S. terrae* DSM 18941^T^	4.41032	143	131	96150	63.90	3670	Soil	LDJJ00000000	This study
19	*S. dokdonesis* DSM 21858^T^	3.55366	34	169	325708	64.50	3063	Soil	LDJL00000000	This study

*^∗^http://genome.jgi.doe.gov/Stema_ATCC_19867/Stema_ATCC_19867.info.html.*

## Methods

### Bacterial Strains and Culture Conditions

Type strains of genus *Stenotrophomonas* and related species (**Table 1**) were procured from different culture collection centers, Microbial Type Culture Collection (MTCC), Belgian Coordinated Collections of Microorganisms/LMG (BCCM/LMG) and The Leibniz Institute DSMZ – German Collection of Microorganisms and Cell Cultures GmbH (DSM). The high molecular weight genomic DNA of *S. africana* LMG 22072 was procured from BCCM/LMG for whole genome sequencing. All isolates were grown as per the media and conditions recommended by the respective culture collection centers.

### Genome Sequencing, Assembly, and Annotation

Genomic DNA was extracted by using ZR Fungal*/*Bacterial DNA MiniPrep Kit (Zymo Research Corporation, Irvine, CA, USA) and quantified using *Qubit* 2.0 Fluorometer (Thermo Fisher Scientific, Waltham, MA, USA). Illumina sequencing library of genomic DNA was prepared using Nextera XT sample preparation kit (Illumina, Inc., San Diego, CA, USA) with dual indexing adapters. Illumina sequencing library was sequenced using in-house Illumina Miseq (Illumina, Inc., San Diego, CA, USA) platform using paired-end sequencing kits. The Illumina adapters were trimmed by the internal software during the base calling. In addition, to that adapter contamination identified by NCBI during the submission was removed by manual trimming. Raw reads were assembled using CLC Genomics Workbench v7.5 (CLC Bio-Qiagen, Aarhus, Denmark) and annotation was using NCBI Prokaryotic Genome Annotation Pipeline through NCBI^2^. CRISPR was identified using the CRISPR recognition tool (Bland et al., 2007).

### Genome Similarity Assessment

For genome similarity assessment we used BLAST-based average nucleotide identity (ANIb) and Genome to Genome Distance calculator or digital DNA-DNA hybridization (dDDH) values. Pairwise ANI was calculated using JSpecies (Richter and Rosselló-Móra, 2009) and digital DDH (Auch et al., 2010) was calculated using web tool GGDC 2.0^3^. *Xanthomonas campestris* pv. *campestries* ATCC 33913 and *P. aeruginosa* DSM 50071 were included as outgroups.

### Pan-Genome Analysis

Pan-genome analysis of representatives of the genus *Stenotrophomonas* and related species under study was carried out by using the PGAP pipeline version 1.12 (Zhao et al., 2012). The MultiParanoid (MP) method was used for Pan-genome analysis with minimum score value 40 and *e*-value e1 × 10^-10^ used as cut off for BLAST. Pan-genome was visualized by using PanGP (Zhao et al., 2014). The flower pot diagram to represent the core and unique genes was drawn by using python script of Matplotlib (Hunter, 2007).

### Data Deposition

The genome sequence data of the 16 type strains sequenced under this study has been deposited in NCBI GenBank and their accession numbers are mentioned in **Table 1**. Single point access to download genomes in FASTA format is also available at Figshare^4^.

### Interpretation of Data Set

#### Genome Sequences and Phylogenic Inference

The general features of the newly sequenced genomes of type strains and their assembly statistics are summarized in **Table 1**. The ANI and dDDH values of representatives of the genus *Stenotrophomonas* are summarized in Supplementary Table S1. In microbial taxonomy for species delineation, 94 and 70% cut-off is used for ANI values and dDDH values, respectively, (Richter and Rosselló-Móra, 2009; Auch et al., 2010). No two strains have >94% ANI and >70% dDDH values suggesting that all the 18 members belong to distinct species.

*Stenotrophomonas africana* was initially described as a novel species but later reclassified as a synonym of *S. maltophilia.* Interestingly ANI and dDDH values of *S. africana* with the type strain of *S. maltophilia* are 90 and 49%, respectively, suggesting that the *S. africana* is a separate species.

The taxonomic status of the misclassified species *P. genicualata, P. hibsicola*, and *P. betele* is unclear and they are considered as synonyms of the *S. maltophilia.* But based on ANI and dDDH values with the type strain of the *S. maltophilia*, these species *P. genicualata, P. hibsicola*, and *P. betele* do not belong to *S. maltophilia* and represent separate species.

*Pseudomonas pictorium* is also considered as a misclassified *Pseudomonas* and is closely related to *Stenotrophomonas* sp. It shows <94% ANI and <70% dDDH with type strains of all *Stenotrophomonas* species and should be reclassified as a distinct species of the genus *Stenotrophomonas*.

*Stenotrophomonas dokdonensis* which was transferred to a new genus *Pseudoxanthomonas* exhibits ANI in the range of 73–75% and dDDH around 20% with the type strain of the species of *Stenotrophomonas*. Hence, there is need to re-examine its classification into a separate genus.

#### Pangenome Analysis of the Genus *Stenotrophomonas*

**FIGURE 1:**
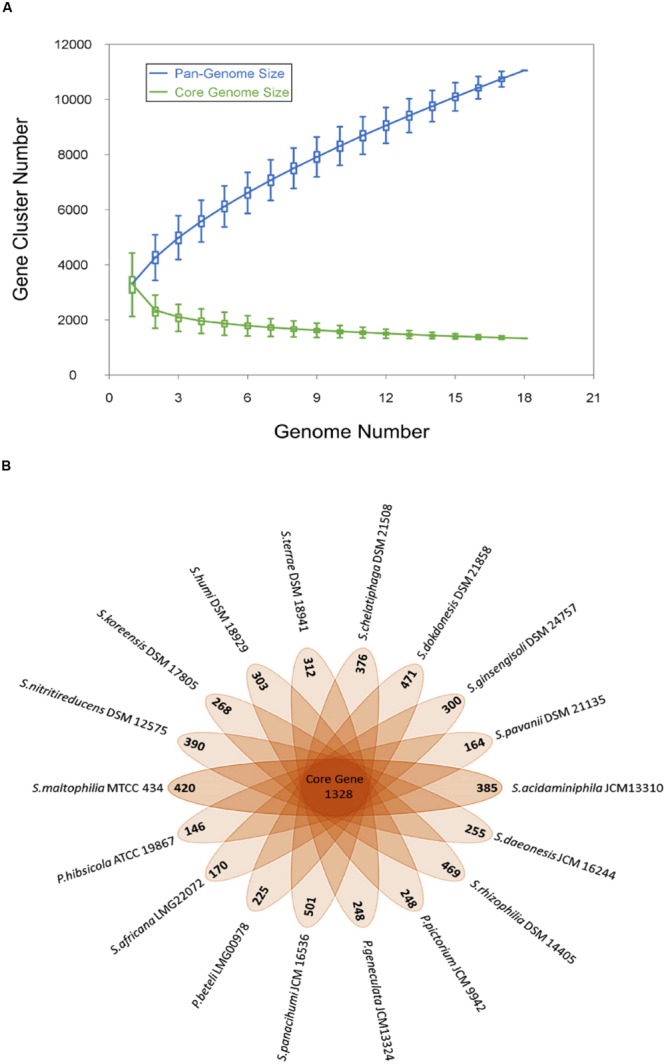
**Pan and core genome of the genus *Stenotrophomonas*. (A)** The number of gene clusters in pan-genome and core-genome are plotted against number of *Stenotrophomonas* genomes sequenced. **(B)** Flower plot diagram showing numbers of unique genes in each *Stenotrophomonas* species in the petals and *Stenotrophomonas* core orthologous gene number in the center.

Genome sequences of the 18 *Stenotrophomonas* species under study were used to analyze the pan-genome and core genome. A total of 11052 genes were identified and among them, 1328 (12%) genes make the core genome of genus *Stenotrophomonas.* The pan and core genome sizes were plotted against the number of genomes under study. The pan-genome curve shows that the power trend line has not attended the plateau (**Figure 1**) suggesting that *Stenotrophomonas* displays an open pan-genome and that the number of genomes analyzed here is not sufficient to describe the complete gene repertoire and it requires more sequencing in order to describe all genes of the genus. The remaining 9724 gene clusters make the accessory genome which also includes the species-specific unique genes that range from 146 to 501 genes (**Figure 1**).

#### Distribution of Chromosomal Metallo-β-Lactamase in Genus *Stenotrophomonas*

Type species of the genus *Stenotrophomonas*, i.e., *S. maltophila* has two chromosomally encoded β-lactamases, L1 and L2 which are characteristics of *S. maltophilia* and gives resistance to almost all β-lactam group of antibiotics (Denton and Kerr, 1998). L1 is a metallo-β-lactamase (Walsh et al., 1994; Ullah et al., 1998) and L2 is a clavulanic acid sensitive serine β-lactamases (Walsh et al., 1997). Here in we accessed the distribution of the L1 metallo-β-lactamase in the representative of the species of the genus. L1 metallo-β-lactamase is exclusively present only in the S. *africana, S. pavani, P. genicualata, P. hibsicola*, and *P. betele* along with the *S. maltophilia* and absent in the other species of the genus. The G+C content of the L1 metallo-β-lactamase is same as that of the genomic GC content, i.e., 67% suggesting that it is not acquired through the lateral gene transfer.

#### CRISPR-*cas* System in *Stenotrophomonas*

CRISPR-*cas* system is important to the bacteria for the adaptive immunity against the invasive elements in bacteria (Barrangou et al., 2007). Among 18 genomes analyzed representing 12 validated species and newly identified species of genus *Stenotrophomonas*, CRISPR loci are absent in the *S. humi, S. koreensis, S. chelatiphaga, S. dokdonesis, S. daeonesis, P. pictorium, S. panacihumi, P. hibsicola* and *P. geniculata*. Interestingly *S. terrae*, and *S. acidaminiphila* have multiple distinct CRISPR locus. The distribution of CRISPR locus across *Stenotrophomonas* genus along with repeat sequences and a number of repeats is mentioned in the Supplementary Table S2. It is important to note that the CRISPR loci are not widespread in this genus as 9 out of 18 genomes of the representative of the genus *Stenotrophomonas* are not having any CRISPR repeats.

## Author Contributions

PP and SM carried out whole genome sequencing and prepared sequin files for submissions. PP and SK carried out the genome analysis. PP drafted the manuscript. PBP conceived the study and participated in its design and coordination. All authors have read and approved the manuscript.

## Conflict of Interest Statement

The authors declare that the research was conducted in the absence of any commercial or financial relationships that could be construed as a potential conflict of interest.

## Footnotes

1 http://www.bacterio.net/stenotrophomonas.html

2 http://www.ncbi.nlm.nih.gov/genome/annotation_prok

3 http://ggdc.dsmz.de/distcalc2.php

4 https://figshare.com/s/7cbcaa37451dab19563d
