# Supplementary material for: Genome Sequence of Type Strains of Genus Stenotrophomonas
Source: Front Microbiol. 2016 Mar 10;7:309. doi: 10.3389/fmicb.2016.00309 (PMC4785145; doi:10.3389/fmicb.2016.00309)
Supplement: Supplementary file 1 [file Data_Sheet_1.DOCX]

Supplementary Material

**Genome sequence of type strains of genus *Stenotrophomonas***

**Prashant P Patil, Samriti Midha, Sanjeet Kumar, Prabhu B Patil***

Bacterial Genomics and Evolution Laboratory, CSIR-Institute of Microbial Technology, Chandigarh, India

***Correspondence:** Dr. Prabhu B Patil, pbpatil@imtech.res.in

**Supplementary Table S1**: **Digital DNA-DNA Hybridization (dDDH) values (in lower triangle) and Average Nucleotide Identity (ANI) values (in upper triangle) amongst different species of *Stenotrophomonas*.**

|  | ***S. maltophilia* MTCC 434** | ***S. africana* LMG22072** | ***P. hibscicola* ATCC 19867** | ***P. beteli* LMG 978** | ***S. pavanii* DSM 25135** | ***P. geniculata* JCM 13324** | ***S. chelatiphaga* DSM 21508** | ***S. rhizophila* DSM 14405** | ***S. panacihumi* JCM 16536** | ***S. koreensis* DSM 17805** | ***S. ginsengisoli* DSM 24757** | ***S. acidaminiphilia* JCM 13310** | ***S. daeonesis* JCM 16244** | ***P. pictorium* JCM 9942** | ***S. humi* DSM 18929** | ***S. nitritrieducens* DSM 12575** | ***S. terrae* DSM 18941** | ***S. dokdonesis* DSM 21858** | ***X. campestris* ATCC 33913** | ***P. aeruginosa* DSM 50071** |
| --- | --- | --- | --- | --- | --- | --- | --- | --- | --- | --- | --- | --- | --- | --- | --- | --- | --- | --- | --- | --- |
| ***S. maltophilia* MTCC 434** | --- | 90.3 | 90.3 | 90.4 | 85.7 | 90 | 85.7 | 81 | 75.4 | 77.3 | 77.7 | 84.3 | 81.9 | 83.6 | 75.7 | 84.2 | 80.2 | 75.7 | 76.4 | 67.8 |
| ***S. africana* LMG 22072** | 49 | --- | 92 | 90.5 | 90.9 | 92.6 | 81.6 | 81 | 77.8 | 75.6 | 75.8 | 79.3 | 79.5 | 78.3 | 77.8 | 79.4 | 77.9 | 74.8 | 76.3 | 67.9 |
| ***P. hibscicola* ATCC 19867** | 47.3 | 46.4 | --- | 90.7 | 91.4 | 93 | 81.3 | 80.9 | 77.8 | 75.9 | 75.9 | 79.4 | 79.6 | 78.4 | 77.9 | 79.4 | 77.9 | 74.4 | 76.3 | 67.8 |
| ***P. beteli* LMG 978** | 42.2 | 42.1 | 41.8 | --- | 90.9 | 90.4 | 81.6 | 80.9 | 77.8 | 75.8 | 75.8 | 79.4 | 79.6 | 78.3 | 77.7 | 79.5 | 77.8 | 74.6 | 76.4 | 67.9 |
| ***S. pavanii* DSM 25135** | 43.1 | 43 | 44.2 | 42.9 | --- | 90.8 | 81.5 | 81.2 | 78 | 75.8 | 75.9 | 79.5 | 79.6 | 78.4 | 77.8 | 79.6 | 78 | 74.7 | 76.3 | 67.7 |
| ***P. geniculata* JCM 13324** | 48.6 | 48.8 | 51 | 41.3 | 42.4 | --- | 81.3 | 81 | 77.7 | 75.7 | 75.7 | 79.3 | 79.4 | 78.5 | 77.7 | 79.5 | 77.8 | 74.7 | 76.2 | 68 |
| ***S. chelatiphaga* DSM 21508** | 25.6 | 25.6 | 25.3 | 25.6 | 25.6 | 25.5 | --- | 80.7 | 77.6 | 75.7 | 75.7 | 79 | 79 | 78.1 | 77.7 | 79.2 | 77.6 | 74.5 | 76.4 | 67.9 |
| ***S. rhizophila* DSM 14405** | 24.8 | 24.6 | 24.5 | 24.7 | 24.8 | 24.5 | 24.5 | --- | 78.2 | 75.7 | 76.2 | 79.8 | 80 | 79.2 | 78.5 | 79.9 | 78.5 | 75 | 77.1 | 68.1 |
| ***S. panacihumi* JCM 16536** | 22.4 | 22.3 | 22.2 | 22.3 | 22.3 | 22.3 | 21.8 | 22.7 | --- | 75.2 | 75.3 | 79 | 79.3 | 77.5 | 76.7 | 79 | 76.6 | 75 | 78.7 | 67.8 |
| ***S. koreensis* DSM 17805** | 21.4 | 21.1 | 21 | 21.1 | 21 | 21.3 | 20.6 | 21.2 | 20.7 | --- | 83.9 | 76.9 | 77.2 | 76.2 | 75.6 | 77 | 75.9 | 73.4 | 74.7 | 68.3 |
| ***S. ginsengisoli* DSM 24757** | 21.3 | 21.1 | 21.2 | 21.4 | 21.2 | 21.5 | 20.7 | 21.1 | 20.3 | 27.5 | --- | 76.9 | 77.1 | 76.3 | 75.7 | 77.1 | 75.9 | 73.3 | 74.4 | 68 |
| ***S. acidaminiphilia* JCM 13310** | 24 | 23.8 | 23.6 | 23.8 | 23.8 | 23.6 | 22.9 | 23.9 | 22.6 | 21.9 | 21.4 | --- | 86.3 | 82.5 | 81.8 | 89.7 | 81.6 | 75.5 | 77.2 | 68.6 |
| ***S. daeonesis* JCM 16244** | 24.1 | 23.9 | 23.8 | 24 | 23.8 | 23.7 | 23.3 | 24.2 | 23.4 | 22.2 | 21.8 | 31.5 | --- | 82.6 | 82.1 | 86.7 | 82 | 75.6 | 77.2 | 68.7 |
| ***P. pictorium* JCM 9942** | 23.1 | 22.6 | 22.4 | 22.6 | 22.7 | 22.7 | 22 | 23 | 21.6 | 21 | 20.9 | 26.3 | 26.5 | --- | 82.3 | 83.4 | 82.8 | 74.5 | 76.5 | 67.8 |
| ***S. humi* DSM 18929** | 22.8 | 22.8 | 22.4 | 22.6 | 22.8 | 22.4 | 22.1 | 22.8 | 21.5 | 21.2 | 21.1 | 25.9 | 26 | 26.1 | --- | 81.9 | 83 | 74.1 | 75.9 | 67.8 |
| ***S. nitritrieducens* DSM 12575** | 24.1 | 24.1 | 23.8 | 24 | 23.9 | 23.9 | 23.4 | 23.9 | 23.1 | 22.1 | 21.8 | 39.4 | 32.3 | 27.4 | 26.3 | --- | 81.8 | 75.5 | 77.5 | 68.1 |
| ***S. terrae* DSM 18941** | 22.8 | 22.6 | 22.4 | 22.6 | 22.7 | 22.7 | 22.3 | 23.2 | 21.7 | 21.3 | 21.2 | 25.6 | 26.2 | 26.7 | 26.8 | 26.1 | --- | 74.3 | 75.8 | 67.1 |
| ***S. dokdonesis* DSM 21858** | 20.4 | 20.6 | 20.4 | 20.3 | 20.6 | 20.1 | 20.1 | 20.7 | 20.2 | 19.9 | 19.5 | 20.6 | 21 | 20.1 | 20.1 | 20.9 | 20.1 | --- | 74.2 | 67.2 |
| ***X. campestris* ATCC 33913** | 21.9 | 21.6 | 21.8 | 21.9 | 22.2 | 21.9 | 21.5 | 22.2 | 23 | 20.4 | 20.3 | 22.1 | 22.2 | 21.5 | 21.4 | 22.2 | 21.4 | 20.6 | --- | 67.4 |
| ***P. aeruginosa* DSM 50071** | 18.1 | 17.9 | 18 | 18.1 | 17.9 | 17.6 | 18 | 18.4 | 17.8 | 18 | 17.5 | 17.9 | 17.7 | 18.3 | 18.2 | 17.8 | 18.4 | 18.6 | 18.6 | --- |

**Supplementary Table S2: Distribution of CRISPR-*cas* system in *Stenotrophomonas*.**

| **Species** | **CRISPR Repeat Sequence** | **Length (bp)** | **Number of Repeats** |
| --- | --- | --- | --- |
| *S. maltophilia* MTCC 434^T^ | AAGAAGGCGGTGAAGAAGG | 19 | 8 |
| *S. africana* LMG 22072 ^T^ | CCTTCTTCGCTGCCTTCTT | 19 | 7 |
| *P. hibsicola* ATCC 19867 ^T^ | None |  |  |
| *P. beteli* LMG 00978 ^T^ | AAGAAAGCGGTGAAGAAGG | 19 | 8 |
| *S. pavanii* DSM 25135 ^T^ | AAGGCGACCAGCAAGAAGGC | 20 | 6 |
|  | AAGAAGGCGGTGAAGAAGG | 19 | 8 |
| *P. geneculata* JCM *13324* ^T^ | None |  |  |
| *S. chelatiphaga* DSM 21508 ^T^ | None |  |  |
| *S. rhizophilia* DSM 14405 ^T^ | TAGTGCCGGCCGCTGGCCG | 19 | 3 |
| *S. panacihumi* JCM 16536 ^T^ | None |  |  |
| *S. koreensis* DSMZ 17805 ^T^ | None |  |  |
| *S. ginsengisoli* DSM 24757 ^T^ | GTTTCAATCCACGCGCCCGCGTGGGGCGCGAC | 32 | 84 |
| *S. acidaminiphila* JCM 13310 ^T^ | AAGGCGGTGAAGAAGGTCGC | 20 | 9 |
|  | GTTCCCTGCCGCATAGGCAGCTCAGAAA | 28 | 35 |
| *S. daeonesis* JCM *16244* ^T^ | None |  |  |
| *P. pictorium* JCM 9942 ^T^ | None |  |  |
| *S. humi* DSMZ 18929 ^T^ | None |  |  |
| *S. nitritireducens* DSMZ 12575^T^ | TTTCTGAGCTGCCCACTCGGCAGCGAAC | 28 | 101 |
| *S. terrae* DSMZ 18941 ^T^ | GTAGTGCCGAGCCATGCTCGGCA | 23 | 4 |
|  | GTAGTGCCGAGCCATGCTCGGCA | 23 | 4 |
| *S. dokdonesis* DSM 21858 ^T^ | None |  |  |
